# Smoking and Coronary Atherosclerosis: Disproportionate Impact on the Right Coronary Artery

**DOI:** 10.1016/j.jscai.2025.103609

**Published:** 2025-05-13

**Authors:** Axel Dahlgren, David Erlinge, Ryo Torii, Enhui Yong, Göran Bergström, Tomas Jernberg, Ole Fröbert, Pernille G. Thrane, Michael Mæng, Gregg W. Stone, Moman A. Mohammad

**Affiliations:** a Department of Cardiology, Clinical Sciences, Lund University, Skåne University Hospital, Lund, Sweden; b Department of Mechanical Engineering, Faculty of Engineering Science, University College London, London, United Kingdom; c Institute of Cardiovascular Science, University College London, London, United Kingdom; d Department of Molecular and Clinical Medicine, Institute of Medicine, Sahlgrenska Academy, University of Gothenburg, Gothenburg, Sweden; e Department of Clinical Physiology, Sahlgrenska University Hospital, Region Västra Götaland, Gothenburg, Sweden; f Division of Cardiovascular Medicine, Department of Clinical Sciences, Karolinska Institutet, Danderyd University Hospital, Stockholm, Sweden; g Department of Cardiology, Faculty of Health, Örebro University, Örebro, Sweden; h Department of Clinical Medicine, Aarhus University Health, Aarhus, Denmark; i Department of Cardiology, Aarhus University Hospital, Aarhus, Denmark; j Icahn School of Medicine at Mount Sinai, New York, New York

**Keywords:** coronary artery atherosclerosis, natural progression, smoking

## Abstract

**Background:**

We aimed to study the long-term effect of smoking on coronary atherosclerosis progression at the segmental level.

**Methods:**

Angiographic data (1989-2017) on current, former, and nonsmokers were collected from the Swedish Coronary Angiography and Angioplasty Registry. The Western Denmark Heart Registry was used to validate the results. Patients with clinically indicated angiography with ≥2 coronary arteries without obstructive coronary artery disease were included. The main outcome was segmental plaque progression, percutaneous coronary intervention, or coronary artery bypass grafting within 15 years.

**Results:**

In total, 215,364 Swedish patients with 993,405 coronary arteries (left anterior descending artery [LAD], left circumflex artery [LCX], and right coronary artery [RCA]) were included. The validation cohort consisted of 19,613 patients. Per 1000 patient-years, plaque progression incidence rate was 11.3 (95% CI, 10.9-11.7) for smokers, 10.2 (95% CI, 9.9-10.5) for former smokers, and 7.7 (95% CI, 7.5-7.9) for nonsmokers. Smokers demonstrated higher relative risk of plaque progression in RCA (hazard ratio, 1.87; 95% CI, 1.73-2.03) vs LAD (hazard ratio, 1.21; 95% CI, 1.12-1.30). Swedish and Danish smokers with ST-segment elevation myocardial infarction had higher proportion of RCA as the culprit artery compared to nonsmokers (smokers: RCA, 42.4%; LAD, 42.0%; LCX, 15.6%; nonsmokers: RCA, 33.1%; LAD, 51.4%; LCX, 15.5%).

**Conclusions:**

This observational cohort study identifies distinct differences in plaque progression patterns between smokers and nonsmokers, with smoking linked to increased plaque progression in the RCA, in contrast to the LAD in nonsmokers. These findings reemphasize the need for targeted smoking prevention and warrant further investigation into RCA-specific mechanisms of plaque progression and MI.

## Introduction

Tobacco smoking is a major public health concern accounting for 7 million deaths per year.^1^ The prevalence of daily tobacco smoking is estimated to be 32.6% for men and 6.5% for women globally and, despite a trend for a decreasing proportion of use, population growth has resulted in a significant increase in the absolute number of smokers over the last several decades.^1^^,^^2^ Smoking is a well-established modifiable risk factor linked to the presence of coronary artery disease (CAD), attributed to the activation of multiple pathophysiological mechanisms involving inflammation, disturbed lipid metabolism, endothelial dysfunction, and increased thrombogenicity.^3^, ^4^, ^5^, ^6^, ^7^, ^8^, ^9^, ^10^, ^11^, ^12^ Although cross-sectional studies have established a higher prevalence of CAD as well as an increased incidence of myocardial infarction (MI) among smokers, there is a lack of robust data on the effect of smoking on plaque development in the coronary tree. Small, cross-sectional studies have either shown no significant spatial differences in CAD distribution or have reported conflicting results, with some reports showing that smoking predominantly affects the left coronary artery whereas others suggest a great impact on the right coronary artery (RCA).^13^, ^14^, ^15^, ^16^ There are also reports of smoking being correlated with inferior myocardial wall infarction.^17^, ^18^, ^19^

We have previously used national cardiac care registries to study the natural history of coronary atherosclerosis.^20^ The complete coverage of all new angiographies in Sweden by the Swedish Coronary Angiography and Angioplasty Registry (SCAAR) provides a unique opportunity to find all new, clinically relevant coronary stenosis progressions resulting in a repeat angiogram. We, therefore, conducted a national register-based study where we identified coronary plaque progression in patients with an index angiography with at least 2 coronaries without stenosis who underwent a clinically indicated repeat angiogram.^20^ The primary objective was to investigate how smoking impacts the segmental progression of coronary artery stenoses using longitudinal data from comprehensive, nationwide coronary angiography registries.

## Methods

### Data sources, subject cohorts, and study design

Data were collected from SCAAR, a nationwide registry established in 1989 in which procedural data on stenosis severity in all investigated coronary artery segments, medical treatments, and medical device use from all coronary angiographies and percutaneous coronary interventions (PCI) performed in Sweden are recorded. SCAAR is a subregistry of the Swedish Web-system for Enhancement and Development of Evidence-Based Care in Heart Disease Evaluated According to Recommended Therapies (SWEDEHEART). Patients included in the registry and each procedure are assigned a unique identification number linked to each Swedish citizen's social security number to make longitudinal follow-up research possible.^21^ The dataset is legally restricted due to Swedish patient privacy and secrecy laws and is therefore unavailable without application to the Uppsala Clinical Research Center.

The present aim was to study the effects of smoking on the progression in coronary artery segments with either no atherosclerosis or subclinical atherosclerosis (defined as <50% stenosis at index angiography). Inclusion and exclusion criteria were crafted accordingly and are available in Supplemental Figure S1. All patients with a first-time angiography performed and recorded in SCAAR between 1989 and 2017 were included regardless of indication (n = 520,824 patients). Patients with ≥2 vessels with segments with ≥50% luminal diameter obstruction (multivessel disease) were excluded (n = 267,060 patients). All branches and segments in the diseased vessel in patients with single-vessel disease were excluded from all analyses (n = 320,268 segments); however, the remaining segments and arteries without obstructive CAD in those patients were included in the study. If a segment with a stenotic lesion progressed within the first 90 days (n = 3310 segments) of index angiography, it was excluded to avoid possible lesions missed at first angiography. Finally, all patients with missing smoking status were excluded (n = 33,372 unique patients, accounting for 388,727 segments). Because not all anatomic variations in the coronary artery tree are reflected in the registry, analyses are limited to the following coronary artery segments: proximal left anterior descending (LAD), middle LAD, distal LAD, proximal RCA, middle RCA, distal RCA, left main coronary artery, proximal left circumflex artery (LCX), distal LCX, first diagonal artery (D1), first obtuse marginal artery (M1), and posterior descending artery/left posterior descending artery. At the artery level, vessels were grouped into 5 major coronary artery segments: RCA (proximal, middle, and distal RCA); LAD (proximal, middle, and distal LAD); LCX (proximal and distal LCX); left main coronary artery; and branches (D1, M1, posterior descending artery/left posterior descending artery).

Each patient and their corresponding coronary artery or segment were tracked longitudinally. If a second clinically indicated coronary angiography was performed, the presence of new coronary stenoses was recorded. Patients who did not undergo a new angiography were considered to have no known coronary plaque progression in this study. Follow-up aimed to evaluate the advancement of nonobstructive coronary artery segments from the initial angiography to the development of obstructive lesions (defined as ≥50% luminal obstruction). Analyses were performed at the patient-level, artery level, and segment level. Patients were followed until the occurrence of ≥50% stenosis, treatment with coronary intervention (PCI or coronary artery bypass grafting [CABG]), death, the conclusion of the 15-year follow-up, or the end of study follow-up, at which point data were censored. If a patient had an obstructive lesion in an artery or segment on a subsequent angiogram, that patient’s other unobstructed arteries and segments remained in the artery and segment level analyses until a new coronary angiogram was performed showing a coronary event, death, or the conclusion of the 15-year follow-up period or end of study follow-up.

Smoking status was defined, at index angiography, as reported smoking habits by the patients registered in SCAAR and the coronary care unit registry from SWEDEHEART. Patients were considered actively smoking even if they had quit within 4 weeks. Patients were divided into cohorts based on smoking status: current smokers, former smokers (> 1-month smoking cessation), and nonsmokers (lifetime nonsmokers) at index angiography. Other risk factors such as hypertension, diabetes mellitus, and hyperlipidemia were attributed if the patient was on medical treatment for the specific risk factor. This study adheres to the Strengthening the Reporting of Observational Studies in Epidemiology (STROBE) guidelines (Supplemental Table S1) and was approved by the Regional Ethical Review Board in Lund, Sweden (Approval ID: 2015/297).

### Outcomes

The primary outcome was progression of coronary artery stenosis from nonobstructive to obstructive detected by angiography, defined as increasing segmental luminal obstruction from <50% to ≥50%, as assessed by the interventional cardiologist, or any stenosis treated with coronary intervention (ie, PCI or CABG).

### Validation analysis of artery level findings patients with ST-segment elevation myocardial infarction

To validate the longitudinal finding of a selective effect of smoking on the RCA, we performed a cross-sectional study and identified the target vessel treated by primary PCI in patients presenting with first-time ST-segment elevation myocardial infarction (STEMI) according to smoking status in Sweden using the SCAAR registry and in Denmark using data from the Western Denmark Heart Registry (WDHR).^22^^,^^23^

### Statistical analysis

Event rates of plaque progression were calculated with the Kaplan-Meier estimator and the incidence rate (IR) with 95% CI per 1000 patient-years, artery-years, or segment-years, depending on the level of analysis. Unadjusted as well as adjusted Cox regression models were applied to estimate hazard ratios (HR) of smoking as a risk factor. In all analyses, nonsmokers were used as the reference. The multivariable analyses were adjusted for age, sex, and risk factors associated with CAD progression (hypertension, hyperlipidemia, and diabetes) if it was not stratified on the same variable. Additional patient-level analyses were stratified by risk factors (hypertension, hyperlipidemia, diabetes, and previous CAD) to evaluate the combined effect of smoking and coexisting risk factors. Additional patient-level analyses were also performed based on an indication at follow-up angiography detecting progressive lesions: chronic coronary syndrome (stable angina), acute coronary syndrome (ACS: STEMI/non-STEMI (NSTEMI)/unstable angina), or other. Additional segment level analyses stratified by coronary artery segments were added (Supplemental Table S2). A post hoc interaction analysis was performed to investigate whether the relative risk of progression in women and men was different (Supplemental Table S3). The proportion of missing data in the original study was overall low; 3.9% for the concurrent risk factors in current smoker vessels was the highest proportion of missing data (Supplemental Table S2). Incidence rates were calculated on complete case data. Statistical analyses were performed using Stata version 17.0 for Macintosh (StataCorp). A 2-sided *P* < .05 was considered significant.

## Results

### Baseline characteristics

A total of 215,364 patients met the inclusion and exclusion criteria and collectively contributed data on 993,405 major coronary arteries (Central Illustration); 18.5% were current smokers, 33.4% were former smokers, and 48.1% were nonsmokers (Table 1). Current smokers (median age, 52 years; IQR, 52-66 years) were younger at the time of index coronary angiography compared with former smokers (median age, 66 years; IQR, 59-72 years) and nonsmokers (median age, 66 years; IQR, 56-74 years). In the overall cohort, 59.2% of the patients were men, with a higher proportion of men among current smokers at 64.8%. Current smokers had more risk factors. Hypertension was prevalent in 46.6% of nonsmokers and in 37.1% of current smokers. Hyperlipidemia was the most common risk factor among all groups, especially in current smokers being 71.8%, compared with former smokers 64.9% and nonsmokers 57.5%. Chronic obstructive pulmonary disease was more prevalent in current smokers. A total of 15.2% of patients underwent at least 1 clinically indicated repeat angiography during the study period and the mean number of angiograms performed per individual was 1.24 for nonsmokers, 1.28 for former smokers, and 1.32 for current smokers (Supplemental Tables S4 and S5). A total of 44.3% of current smokers at baseline who underwent a follow-up coronary angiography were categorized as former smokers on their last follow-up angiography (Supplemental Table S6). The median time to angiography detecting a progressive lesion was 4.5 years (IQR, 1.8-7.2 years) (Supplemental Table S7).

**Table 1:** Baseline characteristics.

Characteristics^a^	Nonsmokers (n = 103,584 patients; n = 482,598 vessels)	Former smokers (n = 71,964 patients; n = 332,134 vessels)	Current smokers (n = 39,816 patients; n = 178,673 vessels)	Total (N = 215,364 patients; N = 993,405 vessels)
Age, y	66 (56-74)	66 (59-72)	59 (52-66)	64 (56-72)
Sex^b^				
Men, patients	56,260 (54.3%)	46,601 (64.8%)	24,627 (64.8%)	127,488 (59.2%)
Men, vessels	258,232 (53.5%)	212,215 (63.9%)	109,545 (61.3%)	579,992 (58.4%)
Women, patients	47,324 (45.7%)	25,363 (35.2%)	15,189 (38.2%)	87,866 (40.8%)
Women, vessels	224,366 (46.5%)	119,919 (36.1%)	69,128 (38.7%)	413,413 (41.6%)
No. of angiograms	1.24 ± 0.65	1.28 ± 0.72	1.32 ± 0.80	1.27 ± 0.70
Follow-up time	5.8 (2.9-9.6)	5.4 (2.7-8.9)	6.1 (3.1-9.9)	5.7 (2.8-9.5)
Hypertension				
Patients	48,257 (46.6%)	37,505 (52.1%)	14,764 (37.1%)	100,526 (46.7%)
Vessels	224,762 (46.6%)	173,149 (52.1%)	66,780 (37.4%)	464,691 (46.8%)
Missing (patients)	666 (0.6%)	453 (0.6%)	469 (1.2%)	1588 (0.7%)
Missing (vessels)	3076 (0.6%)	2074 (0.6%)	2051 (1.2%)	7201 (0.7%)
Hyperlipidemia				
Patients	59,591 (57.5%)	46,719 (64.9%)	28,594 (71.8%)	134,904 (62.6%)
Vessels	269,146 (55.8%)	210,560 (63.4%)	124,957 (69.4%)	604,663 (60.1%)
Missing (patients)	2724 (2.6%)	1433 (2%)	1041 (2.6%)	5198 (2.4%)
Missing, (vessels)	13,213 (2.7%)	6894 (2.1%)	5016 (2.8%)	25,123 (2.5%)
Diabetes				
Patients	12,427 (12%)	10,725 (14.9%)	4475 (11.2%)	27,627 (12.8%)
Vessels	57,205 (11.9%)	49,088 (14.8%)	20,077 (11.2%)	126,370 (12.7%)
Missing (patients)	237 (0.2%)	202 (0.3%)	241 (0.6%)	680 (0.3%)
Missing (vessels)	1059 (0.2%)	907 (0.3%)	1056 (0.6%)	3022 (0.3%)
COPD				
Patients	1711 (1.65%)	5651 (7.9%)	3372 (8.5%)	10,734 (5.0%)
Vessels	8019 (1.7%)	26,402 (8.0%)	15,515 (8.7%)	49,936 (5.0%)
Single-vessel disease				
Patients	43,479 (42%)	33,890 (47.1%)	24,377 (61.2%)	101,746 (47.2%)
Vessels	182,283 (37.8%)	141,941 (42.7%)	101,611 (56.9%)	425,835 (42.9%)
Heart failure				
Patients	6083 (5.9%)	5041 (7.0%)	1528 (3.8%)	12,652 (5.9%)
Vessels	29,182 (6.1%)	23,975 (7.2%)	7273 (4.1%)	60,430 (6.1%)
No. of risk factors including smoking^c^				
0, patients	20,659 (19.9%)	10,204 (14.2%)	0 (0%)	30,863 (14.3%)
0, vessels	103,262 (21.4%)	51,010 (15.4%)	0 (0%)	154,272 (15.5%)
1, patients	24,013 (23.2%)	15,743 (21.9%)	4924 (12.4%)	44,680 (20.8%)
1, vessels	116,850 (24.2%)	76,597 (23.1%)	24,606 (13.8%)	218,053 (22%)
2, patients	33,473 (32.3%)	24,725 (34.4%)	7032 (17.7%)	65,230 (30.3%)
2, vessels	151,051 (31.3%)	111,898 (33.7%)	33,734 (18.9%)	296,683 (29.9%)
3, patients	18,343 (17.7%)	15,752 (21.9%)	16,959 (42.6%)	51,054 (23.7%)
3, vessels	79,748 (16.5%)	68,379 (20.6%)	73,390 (41.1%)	221,517 (22.3%)
≥4, patients	3802 (3.7%)	3674 (5.1%)	9412 (23.6%)	16,888 (7.8%)
≥4, vessels	15,912 (3.3%)	15,402 (4.6%)	40,016 (22.4%)	71,330 (7.2%)
Missing, patients	3294 (3.2%)	1866 (2.6%)	1489 (3.7%)	6649 (3.1%)
Missing, vessels	15,775 (3.3%)	8848 (2.7%)	6927 (3.9%)	31,550 (3.2%)
Major coronary artery segments				
RCA	95,558 (19.8%)	63,382 (19.1%)	31,357 (17.6%)	190,297 (19.1%)
LAD	79,972 (16.6%)	56,408 (17%)	30,597 (17.1%)	166,977 (16.8%)
LCX	100,139 (20.8%)	68,599 (20.7%)	37,164 (20.8%)	205,902 (20.7%)
LMCA	103,365 (21.4%)	71,791 (21.6%)	39,744 (22.2%)	214,900 (21.6%)
Branches	103,564 (21.5%)	71,954 (21.7%)	39,811 (22.3%)	215,329 (21.7%)
Total	482,598 (100%)	332,134 (100%)	178,673 (100%)	993,405 (100%)

Values are n (%), mean ± SD, or median (IQR).

COPD, chronic obstructive pulmonary disease; LAD, left anterior descending artery; LCX, left circumflex artery; LMCA, left main coronary artery; RCA, right coronary artery.

a Percent summing in rows.

b Percent summing in columns.

c Risk factors: Smoking, hypertension, diabetes, hyperlipidemia, or single-vessel disease at index angiography.

### Progressive lesions in clinical subgroups

**Figure 1:**
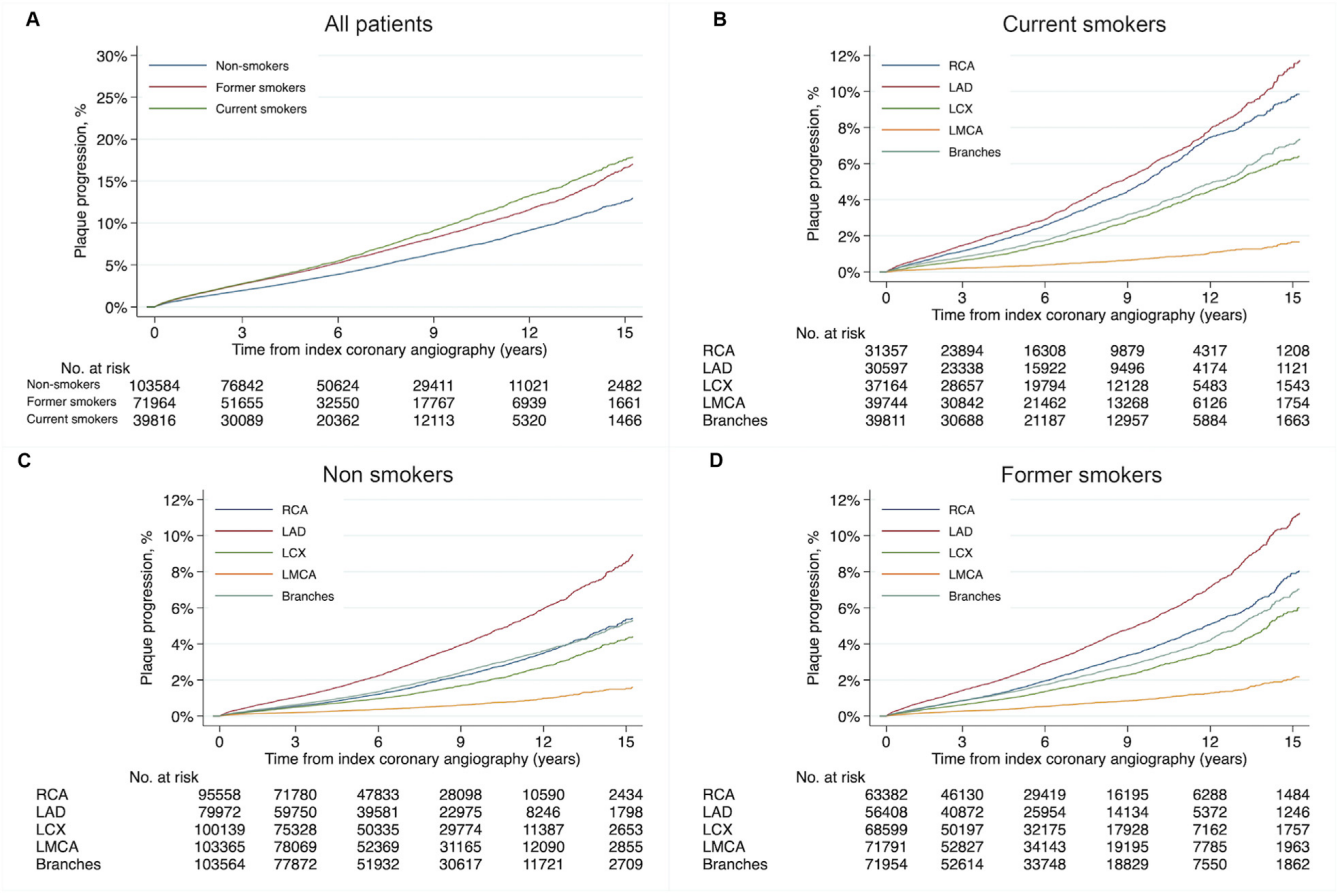
**Fifteen-year progression of coronary atherosclerosis in all patients at the patient-level, and in the major coronary arteries stratified by smoking status.** Kaplan-Meier curves depicting the cumulative probability of progressive lesions ≥50% at the patient and vessel level during the 15-year follow-up time in all patients (**A**) and stratified by major vessels in (**B**) current smokers, (**C**) nonsmokers, and (**D**) former smokers. Branches include segments: the first diagonal, the first obtuse marginal, and the posterior descending left posterior descending artery. LMCA, left main coronary artery;

**Figure 2:**
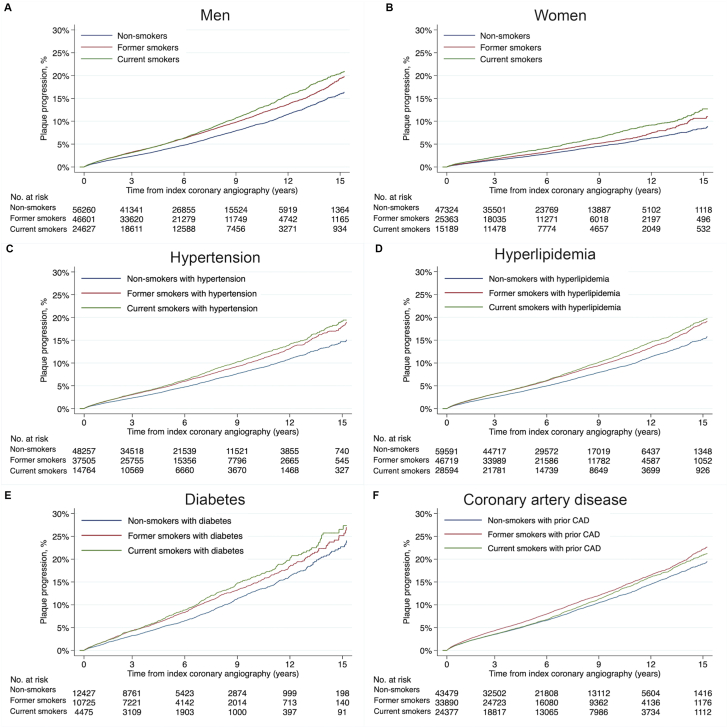
**Patient-level analyses showing the 15-year progression of coronary atherosclerosis in men, women, and patients with other risk factors.** Kaplan-Meier curves depicting the cumulative probability of progressive lesions >50% at the patient-level during the 15-year follow-up time stratified by sex and risk factors associated with CAD progression: (**A**) men, (**B**) women, (**C**) hypertension, (**D**) hyperlipidemia, (**E**) diabetes, and (**F**) previous CAD in a single-vessel.

A total of 18,559 (1.9%) coronary arteries progressed to >50% luminal stenosis or required revascularization by PCI or CABG. Compared with nonsmokers, current smokers had the highest adjusted relative risk of progression with adjusted HR of 1.34 (95% CI, 1.28-1.41; *P* < .001), followed by former smokers, with adjusted HR of 1.16 (95% CI, 1.11-1.21; *P* < .001) (Table 2 and Figure 1). Current smokers had a higher relative risk of progressive lesions leading to ACS compared with nonsmokers, with adjusted HR of 1.52 (95% CI, 1.44-1.62; *P* < .001). Former smokers had an adjusted HR of 1.12 (95% CI, 1.06-1.19; *P* < .001), compared with nonsmokers (Supplemental Table S3). Men demonstrated a higher cumulative incidence of plaque progression than women (Supplemental Table S3, Figure 2). The relative increase associated with smoking was higher for female smokers, with adjusted HR of 1.50 (95% CI, 1.36-1.64; *P* < .001), compared with male smokers with adjusted HR of 1.30 (95% CI, 1.23-1.38; *P* < .001), but the interaction of sex and smoking was not statistically significant when current smoker HR for women and men were compared (adjusted women-to-men HR, 1.08; 95% CI, 0.97-1.20; *P* = .16) (Supplemental Table S3). Adjusted HR was similar among all the traditional risk factors when comparing smokers with nonsmokers (hypertension: 1.27; 95% CI, 1.18-1.37; *P* < .001; hyperlipidemia: 1.20; 95% CI, 1.14-1.27; *P* < .001; diabetes: 1.21; 95% CI, 1.08-1.36; *P <* .001), except for prior CAD in coexistence with smoking where the adjusted HR was insignificant at 0.99 (95% CI, 0.94-1.05; *P* = .90) (Supplemental Table S3, Figure 2).

**Table 2:** Primary outcomes: Incidence rates for new coronary artery lesions defined as ≥50% luminal stenosis, or treatment with CABG or PCI.

Smoking status	Follow-up (person-year)	KM event rate	IR (95% CI)	HR (95% CI)	Adj HR (95% CI)
All patients					
Nonsmoker	633,151	4871 (13.0%)	7.7 (7.5-7.9)	Reference	Reference
Former smoker	416,712	4247 (17.0%)	10.2 (9.9-10.5)	1.33 (1.27-1.39)^a^	1.16 (1.11-1.21)^a^
Current smoker	254,959	2877 (17.9%)	11.3 (10.9-11.7)	1.45 (1.38-1.52)^a^	1.34 (1.28-1.41)^a^
Major coronary artery segments					
RCA					
Nonsmoker	594,795	1581 (5.4%)	2.7 (2.5-2.8)	Reference	Reference
Former smoker	373,425	1484 (8.1%)	4.0 (3.8-4.2)	1.51 (1.41-1.62)^a^	1.31 (1.22-1.41)^a^
Current smoker	203,823	1141 (9.9%)	5.6 (5.3-5.9)	2.07 (1.91-2.23)^a^	1.87 (1.73-2.03)^a^
LAD					
Nonsmoker	491,507	2308 (9.0%)	4.7 (4.5-4.9)	Reference	Reference
Former smoker	329,589	1927 (11.3%)	5.8 (5.6-6.1)	1.25 (1.18-1.33)^a^	1.05 (0.99-1.12), *P = .13*
Current smoker	198,274	1274 (11.7%)	6.4 (6.1-6.8)	1.32 (1.25-1.44)^a^	1.21 (1.12-1.30)^a^
LCX					
Nonsmoker	626,575	1317 (4.4%)	2.1 (2.0-2.2)	Reference	Reference
Former smoker	408,767	1149 (6.0%)	2.8 (2.7-3.0)	1.35 (1.25–1.46)^a^	1.15 (1.06-1.24)^a^
Current smoker	246,629	838 (6.4%)	3.4 (3.2-3.6)	1.57 (1.44–1.72)^a^	1.44 (1.31-1.57)^a^
LMCA					
Nonsmoker	651,507	504 (1.6%)	0.8 (0.7-0.8)	Reference	Reference
Former smoker	432,603	455 (2.2%)	1.1 (1.0-1.2)	1.35 (1.20-1.55)^a^	1.18 (1.04-1.35), *P = .01*
Current smoker	267,123	224 (1.7%)	0.8 (0.7-1.0)	1.06 (0.90-1.24), *P=0.49*	1.03 (0.88-1.22), *P = .69*
Branches					
Nonsmoker	646,575	1835 (5.3%)	2.8 (2.7-3.0)	Reference	Reference
Former smoker	428,767	1494 (7.0%)	2.5 (3.3-3.7)	1.24 (1.15-1.32)^a^	1.07 (0.99-1.14), *P = .08*
Current smoker	264,019	1028 (7.4%)	3.9 (3.7-4.1)	1.34 (1.24-1.4)^a^	1.23 (1.13-1.33)^a^

Table depicting incidence rates per 1000-patient-years/vessel-years, unadjusted hazard ratio, and adjusted hazard ratios, with 95% CI for progressive lesions with ≥50% luminal obstruction at patient and vessel level. The indication is based on an indication at an angiogram detecting the progressive lesion. KM event rate indicates cumulative incidence at the end of follow-up (15 years).

Adj, adjusted; IR, incidence rate; KM, Kaplan-Meier; LAD, left anterior descending artery; LCX, left circumflex artery; LMCA, left main coronary artery; RCA, right coronary artery.

a *P* < .001.

### Progressive lesions in different regions of the coronary tree

The LAD in smokers showed the highest IR per 1000 artery-years (current smokers: 6.4, 95% CI, 6.1-6.8; former smokers: 5.8, 95% CI, 5.6-6.1; nonsmokers: 4.7, 95% CI, 4.5-4.9), and an adjusted HR of 1.21, 95% CI, 1.12 to 1.30; *P* < .001, when comparing current smokers with nonsmokers (Table 1). Although IR for plaque progression were lower in RCA compared to the LAD, smokers had a higher relative risk of plaque progression in RCA with an adjusted HR of 1.87, 95% CI, 1.73 to 2.03; *P* < .001 (Table 1, Figure 1, Supplemental Figure S2). Comparison of adjusted HR in the RCA to other major arteries using interaction terms confirmed significantly higher relative risk in the RCA (Supplemental Table S8).

### Validation analysis of artery level findings in STEMI patients

**Figure 3:**
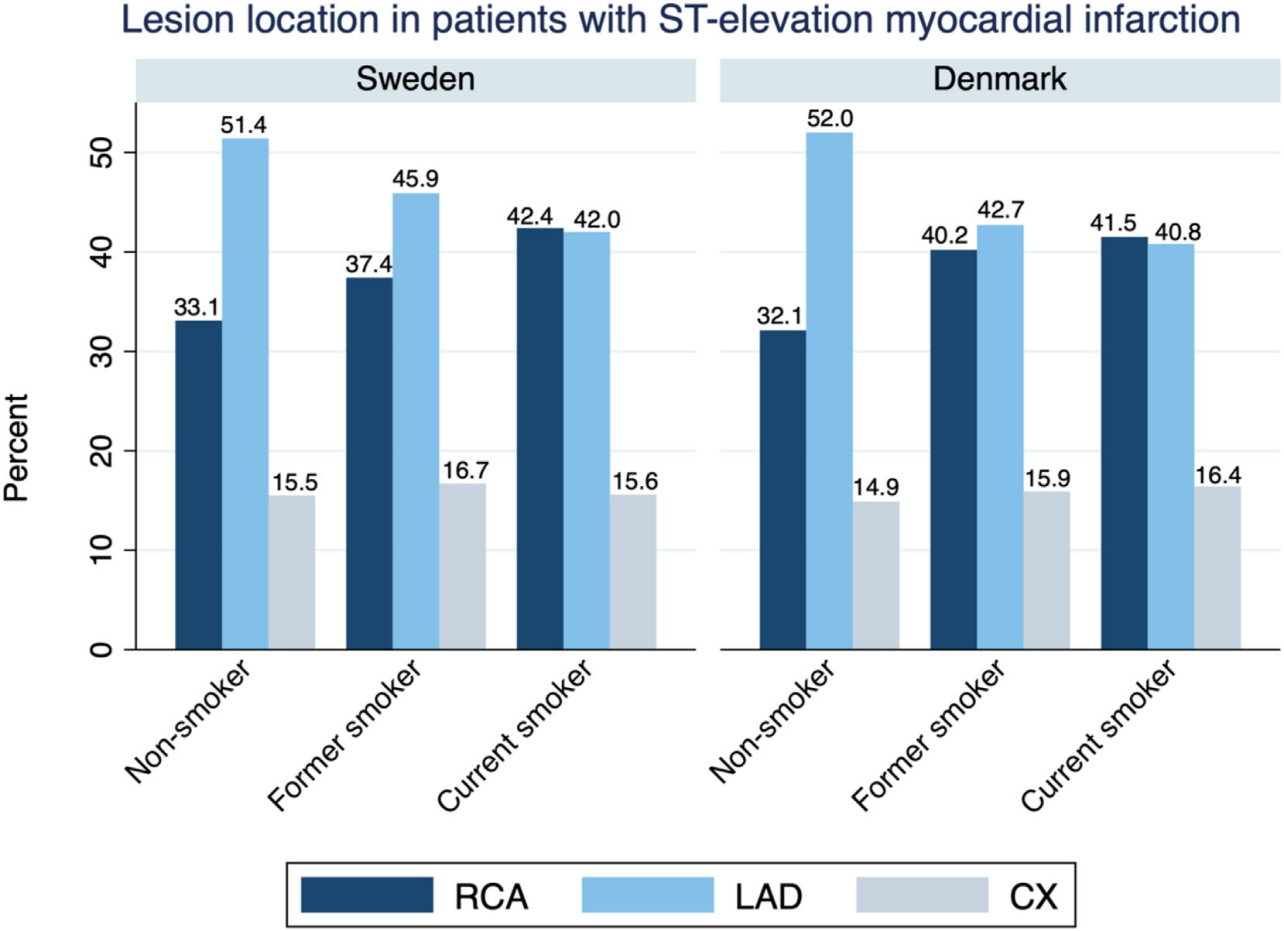
**External validation of results.** Numbers are percentages. Distribution of target vessels in patients presenting with ST-segment elevation myocardial infarction treated with primary percutaneous coronary intervention in Denmark and Sweden. Danish data: a few lesions were unclassified culprit lesions (the sum is not 100%).

From the SCAAR database, we identified patients with first-time primary PCI due to STEMI from 1989 to 2018 and assessed all coronary artery lesions with ≥50% luminal stenosis or lesions treated with primary PCI within the RCA, LAD, and LCX by smoking status. A total of 79,380 lesions in 57,674 patients were identified in SCAAR. A total of 9209 lesions in 6595 patients had missing smoking data and were therefore excluded. The results were then compared with a similar cohort of STEMI patients undergoing first-time primary PCI from January 1, 2003, to October 31, 2018, from the WDHR database. A total of 23,239 lesions in 19,613 patients were identified in WDHR with 5339 lesions in 4428 patients having missing smoking data. After excluding patients with missing smoking data, the total study population consisted of 51,079 and 15,185 patients presenting with STEMI from the SCAAR and WDHR, respectively. In SCAAR, RCA was the most common culprit vessel in current smokers (RCA: 42.4%; LAD 42.0%; LCX: 15.6%) whereas LAD was the most common target vessel in nonsmokers (RCA: 33.1%; LAD: 51.4%; LCX: 15.5%) (Figure 3). This association was confirmed in the WDHR with RCA as the most common culprit vessel in smokers (RCA: 41.5%, LAD: 40.8%, LCX: 16.4%), and LAD the most common culprit vessel in nonsmokers (RCA: 32.1%, LAD: 52%, LCX: 14.9%) (Figure 3).

**Central Illustration:**
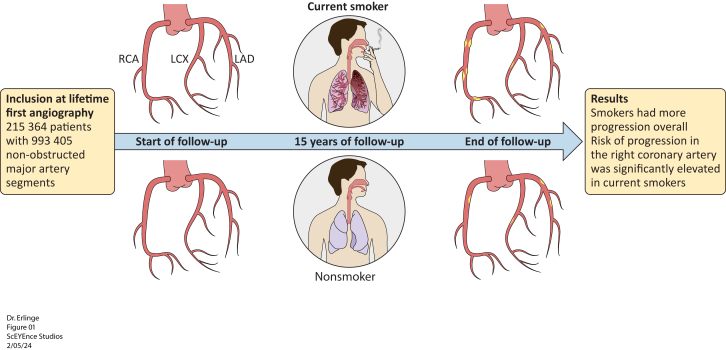
**Illustration of the impact of smoking on coronary artery plaque progression seen on coronary angiography over the 15-year follow-up time.** The top panel depicts progression in smokers characterized by a high progression rate in the LAD and the RCA. The bottom panel depicts progression in nonsmokers characterized by a higher progression rate in the left coronary artery. LCX, left circumflex artery.

## Discussion

In this nationwide long-term study, smoking was associated with increased rates of coronary plaque progression at the arterial and segmental levels. Smoking conferred a comparable excess risk across patients with hypertension, hyperlipidemia, or diabetes. An important novel finding was the disproportionate impact of smoking on the RCA compared with the LAD. Among smokers, the relative risk of plaque progression was higher in the RCA, offsetting the typically greater absolute risk of plaque progression observed in the LAD. This hypothesis was further tested in first-time STEMI patients treated with primary PCI in Sweden and Denmark, demonstrating in both databases a higher incidence of new culprit lesions in the RCA in smokers compared with the LAD in nonsmokers. This finding has implications for understanding the mechanisms of how smoking impacts CAD.

This study is to our knowledge the largest and longest study assessing the impact of smoking on the development of segmental coronary artery lesions by relying on close to complete nationwide assessment of new angiographies. Consistent with previous research, our main finding was that smokers and former smokers were younger at the time of presentation and had a higher overall rate of CAD progression and future risk of ACS.^4^^,^^24^ Previous observations have indicated that smoking might increase the risk of cardiovascular events more in women than in men.^25^^,^^26^ In our study, smoking was associated with a trend toward a higher relative risk of plaque development in women compared with men. However, this association was not significant in the interaction analysis. The augmented effect of smoking on plaque progression together with other risk factors such as diabetes, hyperlipidemia, hypertension, and previous CAD was similar and was an expected finding as traditional risk factors for CAD are known to have additive effects.^27^^,^^28^ In line with previous research, smokers with plaque progression more often presented with ACS compared with nonsmokers.^14^^,^^29^ This could be indicative of higher thrombogenicity in smokers, or due to the higher prevalence of vulnerable plaques among patients who smoke, although previous intravascular imaging studies on plaque composition have yielded results both in favor and against more frequent vulnerable plaque characteristics in smokers.^30^, ^31^, ^32^, ^33^, ^34^

### Arterial and segmental analysis of the coronary tree

Although the LAD was the predominant site of obstructive CAD overall, a shift toward the RCA was observed in smokers. This finding was validated in our cross-sectional study of STEMI patients in whom the proportion of RCA as the culprit artery was higher in smokers compared to nonsmokers. In smokers, the RCA and LAD were equally common as the culprit artery, whereas in nonsmokers, LAD was the predominant culprit artery. The association between smoking and distribution of CAD in the coronary tree has been explored before, albeit not extensively, and with conflicting results. A systematic review of 11 studies with 6037 patients, reported scarce evidence favoring any specific location within the coronary artery tree among smokers.^16^ Koliaki et al^13^ studied coronary angiograms in 1228 patients and reported a similar prevalence of CAD in all 3 major vessels, as did Castela et al^14^ studying 521 patients. A smaller cross-sectional study on 8705 patients by Vander Zwaag et al^15^ indicated a higher relative risk of lesions in the RCA for current smokers compared with nonsmokers. Smoking has also been associated with inferior wall infarcts.^17^, ^18^, ^19^ This finding could add context to the controversial “Smoker’s paradox” suggesting better outcomes after MI in smokers—a phenomenon that has been linked to lower age in smokers.^35^ Because inferior infarcts are associated with better outcomes than anterior infarcts, the RCA predominance in smokers could help explain the smoker’s paradox.^36^

The underlying mechanism driving the smoking-associated predominance of plaque progression in the RCA warrants further investigation. A deeper understanding of this smoking-specific CAD phenotype could pave the way for novel prophylactic treatments. Locally increased thrombogenicity facilitated by the static flow and geometric properties of the RCA has been speculated.^18^^,^^37^^,^^38^ A computational flow modeling study found RCA flow to shift to phasic and diastolic dominant in the setting of pulmonary hypertension.^39^ A shift to phasic flow would contradict the theory of static flow increasing thrombogenicity. Although the role of hemodynamic forces regarding CAD is uncertain, phasic flow is thought to increase atherogenic shear stress and explain local differences in CAD, specifically the propensity of the LAD to develop atherosclerosis in the nonsmoking population.^40^, ^41^, ^42^, ^43^, ^44^, ^45^, ^46^ Thus, altered hemodynamic forces in the RCA due to right ventricular remodeling in smokers facilitating the development of vulnerable plaque could serve as a potential explanation worth exploring in future studies.

### Limitations

This study is subject to several limitations. First and foremost is the lack of data concerning the duration and quantity of smoking. However, the onset of smoking typically occurs in youth, whereas clinical manifestations of CAD usually occur decades later, suggesting an expected long duration and significant pack-year history. Additionally, for individuals classified as former smokers, the precise timeframe of their smoking cessation has been reported to be of importance but is not registered in SCAAR or WDHR.^46^ Second, follow-up data on plaque progression relied on the clinical indication to perform a coronary angiogram. Patients not performing an angiogram were considered as not having plaque progression in this study. As a result, lesions in individuals with asymptomatic progression or with medically treated angina, or in those who died before a potential angiography could not be assessed were not detected, likely contributing to an underestimation of plaque progression. Third, data on obesity, low-density lipoprotein cholesterol, alcohol consumption, and family history of CAD are not captured in SCAAR and may have yielded additional insights. Finally, only coronary angiograms performed in catheterization labs in Sweden, for the original analysis, and Denmark, for the supplementary validation analysis, were included. Emigration rates are low in these countries and the registry coverage is close to 100%.^21^^,^^22^

## Conclusion

This nationwide longitudinal study underscores the link between smoking, accelerated CAD progression, and presentation with ACS. Moreover, our data reveal a higher incidence of progressive lesions in the RCA among smokers compared with nonsmokers, confirmed in STEMI patients. These findings reemphasize the need for targeted smoking prevention and warrant further investigation into RCA-specific mechanisms of plaque progression and MI.

## References

[1] Dai X., Gakidou E., Lopez A.D. (2022). Evolution of the global smoking epidemic over the past half century: strengthening the evidence base for policy action. Tob Control.

[2] Reitsma M.B., Kendrick P.J., Ababneh E., Abbafati C., Abbasi-Kangevari M., Abdoli A. (2021). Spatial, temporal, and demographic patterns in prevalence of smoking tobacco use and attributable disease burden in 204 countries and territories, 1990-2019: a systematic analysis from the Global Burden of Disease Study 2019. Lancet.

[3] Brown J.C., Gerhardt T.E., Kwon E. (Updated June 5, 2022). https://www.ncbi.nlm.nih.gov/books/NBK554410/.

[4] Larsson S.C., Mason A.M., Bäck M. (2020). Genetic predisposition to smoking in relation to 14 cardiovascular diseases. Eur Heart J.

[5] Ding N., Sang Y., Chen J. (2019). Cigarette smoking, smoking cessation, and long-term risk of 3 major atherosclerotic diseases. J Am Coll Cardiol.

[6] Oshunbade A.A., Kassahun-Yimer W., Valle K.A. (2021). Cigarette smoking, incident coronary heart disease, and coronary artery calcification in black adults: the Jackson Heart study. J Am Heart Assoc.

[7] Jha P., Ramasundarahettige C., Landsman V. (2013). 21st-century hazards of smoking and benefits of cessation in the United States. N Engl J Med.

[8] Hackshaw A., Morris J.K., Boniface S., Tang J.L., Milenković D. (2018). Low cigarette consumption and risk of coronary heart disease and stroke: meta-analysis of 141 cohort studies in 55 study reports. BMJ.

[9] Gleerup H.B., Dahm C.C., Thim T. (2020). Smoking is the dominating modifiable risk factor in younger patients with STEMI. Eur Heart J Acute Cardiovasc Care.

[10] Olesen K.K.W., Thrane P.G., Würtz M., Kristensen S.D., Maeng M. (2022). Cardiovascular risks associated with smoking in patients without obstructive coronary artery disease. Eur J Prev Cardiol.

[11] Ambrose J.A., Barau R.S. (2004). The pathophysiology of cigarette smoking and cardiovascular disease: an update. JAMA.

[12] Messner B., Bernhard D. (2014). Smoking and cardiovascular disease: mechanisms of endothelial dysfunction and early atherogenesis. Arterioscler Thromb Vasc Biol.

[13] Koliaki C., Sanidas E., Dalianis N. (2011). Relationship between established cardiovascular risk factors and specific coronary angiographic findings in a large cohort of Greek catheterized patients. Angiology.

[14] Castela S., Duarte R., Reis R.P. (2004). Acute coronary syndromes in smokers: clinical and angiographic characteristics. Rev Port Cardiol.

[15] Vander Zwaag R., Lemp G.F., Hughes J.P. (1988). The effect of cigarette smoking on the pattern of coronary atherosclerosis. A case-control study. Chest.

[16] Salehi N., Janjani P., Tadbiri H., Rozbahani M., Jalilian M. (2021). Effect of cigarette smoking on coronary arteries and pattern and severity of coronary artery disease: a review. J Int Med Res.

[17] Toluey M., Ghaffari S., Tajlil A., Nasiri B., Rostami A. (2019). The impact of cigarette smoking on infarct location and in-hospital outcome following acute ST-elevation myocardial infarction. J Cardiovasc Thorac Res.

[18] Grines C.L., Topol E.J., O'Neill W.W. (1995). Effect of cigarette smoking on outcome after thrombolytic therapy for myocardial infarction. Circulation.

[19] Gomez M.A., Karagounis L.A., Allen A., Anderson J.L. (1993). Effect of cigarette smoking on coronary patency after thrombolytic therapy for myocardial infarction. TEAM-2 Investigators. Second Multicenter Thrombolytic Trials of Eminase in Acute Myocardial Infarction. Am J Cardiol.

[20] Mohammad M.A., Stone G.W., Koul S. (2022). On the natural history of coronary artery disease: a longitudinal nationwide serial angiography study. J Am Heart Assoc.

[21] Jernberg T., Attebring M.F., Hambraeus K. (2010). The Swedish Web-system for Enhancement and Development of Evidence-based care in Heart disease Evaluated According to Recommended Therapies (SWEDEHEART). Heart.

[22] Schmidt M., Maeng M., Madsen M., Sørensen H.T., Jensen L.O., Jakobsen C.J. (2018). The Western Denmark Heart Registry: its influence on cardiovascular patient care. J Am Coll Cardiol.

[23] Thrane P.G., Olesen K.K.W., Thim T. (2023). Mortality trends after primary percutaneous coronary intervention for ST-segment elevation myocardial infarction. J Am Coll Cardiol.

[24] Weisz G., Cox D.A., Garcia E. (2005). Impact of smoking status on outcomes of primary coronary intervention for acute myocardial infarction—the smoker's paradox revisited. Am Heart J.

[25] Huxley R.R., Woodward M. (2011). Cigarette smoking as a risk factor for coronary heart disease in women compared with men: a systematic review and meta-analysis of prospective cohort studies. Lancet.

[26] Higashi S., Shiga Y., Yano M. (2021). Associations between smoking habits and major adverse cardiovascular events in patients who underwent coronary computed tomography angiography as screening for coronary artery disease. Heart Vessels.

[27] Grundy S.M., Pasternak R., Greenland P., Smith S., Fuster V. (1999). Assessment of cardiovascular risk by use of multiple-risk-factor assessment equations: a statement for healthcare professionals from the American Heart Association and the American College of Cardiology. Circulation.

[28] Wilson P.W., D’Agostino R.B., Levy D., Belanger A.M., Silbershatz H., Kannel W.B. (1998). Prediction of coronary heart disease using risk factor categories. Circulation.

[29] Zhang X., Peng X., Li L., Yu H., Yu B. (2021). Persistent cigarette smoking attenuates plaque stabilization in response to lipid-lowering therapy: a serial optical coherence tomography study. Front Cardiovasc Med.

[30] Gambardella J., Sardu C., Sacra C., Del Giudice C., Santulli G. (2017). Quit smoking to outsmart atherogenesis: molecular mechanisms underlying clinical evidence. Atherosclerosis.

[31] Kang S.J., Mintz G.S., Witzenbichler B. (2015). Age-related effects of smoking on culprit lesion plaque vulnerability as assessed by grayscale and virtual histology-intravascular ultrasound. Coron Artery Dis.

[32] Bolorunduro O., Cushman C., Kapoor D. (2015). Comparison of coronary atherosclerotic plaque burden and composition of culprit lesions between cigarette smokers and non-smokers by in vivo virtual histology intravascular ultrasound. J Invasive Cardiol.

[33] Buljubasic N., Akkerhuis K.M., de Boer S.P. (2015). Smoking in relation to coronary atherosclerotic plaque burden, volume and composition on intravascular ultrasound. PLoS One.

[34] Kornowski R. (1999). Impact of smoking on coronary atherosclerosis and remodeling as determined by intravascular ultrasonic imaging. Am J Cardiol.

[35] Andrikopoulos G.K., Richter D.J., Dilaveris P.E. (2001). In-hospital mortality of habitual cigarette smokers after acute myocardial infarction. The "smoker’s paradox" in a countrywide study. Eur Heart J.

[36] Stone P.H., Raabe D.S., Jaffe A.S. (1988). Prognostic significance of location and type of myocardial infarction: independent adverse outcome associated with anterior location. J Am Coll Cardiol.

[37] Barbash G.I., Reiner J., White H.D. (1995). Evaluation of paradoxic beneficial effects of smoking in patients receiving thrombolytic therapy for acute myocardial infarction: mechanism of the “smoker’s paradox” from the GUSTO-I trial, with angiographic insights. Global Utilization of Streptokinase and Tissue-Plasminogen Activator for Occluded Coronary Arteries. J Am Coll Cardiol.

[38] Mølstad P. (1991). First myocardial infarction in smokers. Eur Heart J.

[39] Mynard J.P., Smolich J.J. (2016). Influence of anatomical dominance and hypertension on coronary conduit arterial and microcirculatory flow patterns: a multiscale modeling study. Am J Physiol Heart Circ Physiol.

[40] Goodwill A.G., Dick G.M., Kiel A.M., Tune J.D. (2017). Regulation of coronary blood flow. Compr Physiol.

[41] Giannoglou G., Antoniadis A., Koskinas K., Chatzizisis Y. (2011). Difference in the localisation of coronary artery disease between the left and right coronary artery system. Eur J Cardiovasc Med.

[42] Papafaklis M.I., Takahashi S., Antoniadis A.P. (2015). Effect of the local hemodynamic environment on the de novo development and progression of eccentric coronary atherosclerosis in humans: insights from PREDICTION. Atherosclerosis.

[43] Brown A.J., Teng Z., Evans P.C., Gillard J.H., Samady H., Bennett M.R. (2016). Role of biomechanical forces in the natural history of coronary atherosclerosis. Nat Rev Cardiol.

[44] Shaaban A.M., Duerinckx A.J. (2000). Wall shear stress and early atherosclerosis: a review. AJR Am J Roentgenol.

[45] Zhu H., Friedman M.H. (2003). Relationship between the dynamic geometry and wall thickness of a human coronary artery. Arterioscler Thromb Vasc Biol.

[46] Doll R., Peto R., Boreham J., Sutherland I. (2004). Mortality in relation to smoking: 50 years' observations on male British doctors. BMJ.

