# Supplementary material for: Smoking and Coronary Atherosclerosis: Disproportionate Impact on the Right Coronary Artery
Source: J Soc Cardiovasc Angiogr Interv. 2025 May 13;4(8):103609. doi: 10.1016/j.jscai.2025.103609 (PMC12462097; doi:10.1016/j.jscai.2025.103609)
Supplement: Supplementary Material [file mmc1.docx]

**Supplementary Material**

Smoking and Coronary Atherosclerosis: Disproportionate impact on the right coronary artery
*Axel Dahlgren MD^1^, David Erlinge MD PhD^1^, Ryo Torii PhD^2^, Enhui Yong MD^2,3^, Göran Bergström MD PhD^4,5^, Tomas Jernberg MD PhD^6^, Ole Fröbert MD PhD^7,8^, Pernille Thrane MD^9^, Michael Maeng MD PhD^9^, Gregg W. Stone MD PhD^10^, Moman A. Mohammad MD PhD^1^.*

1. Department of Cardiology, Clinical Sciences, Lund University, Skåne University Hospital, Lund, Sweden

2. Department of Mechanical Engineering, Faculty of Engineering Science, University College London, London, United Kingdom

3. Institute of Cardiovascular Science, University College London, London, UK

4. Department of Molecular and Clinical Medicine, Institute of Medicine, Sahlgrenska Academy, University of Gothenburg, Gothenburg, Sweden

5. Department of Clinical Physiology, Sahlgrenska University Hospital, Region Västra Götaland, Gothenburg, Sweden

6. Division of Cardiovascular Medicine, Department of Clinical Sciences, Karolinska Institutet, Danderyd University Hospital, Stockholm, Sweden

7. Department of Cardiology, Faculty of Health, Örebro University, Örebro

8. Department of Clinical Medicine, Aarhus University Health, Aarhus, Denmark

9. Department of Cardiology, Aarhus University Hospital, Aarhus, Denmark

10. Icahn School of Medicine at Mount Sinai, New York, NY, USA

**Corresponding author:** David Erlinge, Department of Cardiology, Clinical Sciences, Lund University, Skane University Hospital, Lund; 221 85 Lund, Sweden; telephone: +46 704 872194; fax: +46 46 157857; email: [david.erlinge@med.lu.se](mailto:david.erlinge@med.lu.se)

## **Table of Contents**

[Table of Contents 2](#_Toc191487462)

[Supplementary Table 1. STROBE Statement—checklist of items that should be included in reports of observational studies. 3](#_Toc191487463)

[Supplementary Table 2: Incidence rates in proximal, mid or distal coronary arteries, unadjusted hazard ratios, and adjusted hazard ratios for plaque progression ≥50% luminal obstruction, or segment treated with PCI or CABG 5](#_Toc191487464)

[Supplementary Table 3: Primary outcomes stratified by sex, indication, and risk factors: Incidence rates for new coronary artery lesions defined as ≥50% luminal stenosis, or lesion treated with CABG or PCI 7](#_Toc191487465)

[Supplementary Table 4: Mean number of coronary angiographies by smoking status 9](#_Toc191487466)

[Supplementary Table 5: Patients divided by numbers of follow-up angiographies performed and smoking status 10](#_Toc191487467)

[Supplementary Table 6: Smoking status at life-time last follow-up angiography divided by smoking status at baseline angiography 11](#_Toc191487468)

[Supplementary Table 7: Median time to angiography detecting progressive lesion on patient level 12](#_Toc191487469)

[Supplementary Table 8: Adjusted relative risk of progression in major coronary arteries compared with the right coronary artery 14](#_Toc191487470)

[Supplementary Figure 1: Inclusion and exclusion flow chart 15](#_Toc191487471)

[Supplementary Figure 2: Total distribution of progression lesions for each smoking-cohort 16](#_Toc191487472)

# **Supplementary Table 1. STROBE Statement—checklist of items that should be included in reports of observational studies.**

|  | Item No | Recommendation | Page No |
| --- | --- | --- | --- |
| **Title and abstract** | 1 | (*a*) Indicate the study’s design with a commonly used term in the title or the abstract | 3 |
|  |  | (*b*) Provide in the abstract an informative and balanced summary of what was done and what was found | 2-3 |
| Introduction | | |  |
| Background/rationale | 2 | Explain the scientific background and rationale for the investigation being reported | 5 |
| Objectives | 3 | State specific objectives, including any prespecified hypotheses | 5 |
| Methods | | |  |
| Study design | 4 | Present key elements of study design early in the paper | 5-7 |
| Setting | 5 | Describe the setting, locations, and relevant dates, including periods of recruitment, exposure, follow-up, and data collection | 5-7 |
| Participants | 6 | (*a*) *Cohort study*—Give the eligibility criteria, and the sources and methods of selection of participants. Describe methods of follow-up  *Case-control study*—Give the eligibility criteria, and the sources and methods of case ascertainment and control selection. Give the rationale for the choice of cases and controls  *Cross-sectional study*—Give the eligibility criteria, and the sources and methods of selection of participants | 5-7 |
|  |  | (*b*) *Cohort study*—For matched studies, give matching criteria and number of exposed and unexposed  *Case-control study*—For matched studies, give matching criteria and the number of controls per case |  |
| Variables | 7 | Clearly define all outcomes, exposures, predictors, potential confounders, and effect modifiers. Give diagnostic criteria, if applicable | 7-9 |
| Data sources/ measurement | 8* | For each variable of interest, give sources of data and details of methods of assessment (measurement). Describe comparability of assessment methods if there is more than one group | 7-9 |
| Bias | 9 | Describe any efforts to address potential sources of bias | 7-9 |
| Study size | 10 | Explain how the study size was arrived at | 6 |
| Quantitative variables | 11 | Explain how quantitative variables were handled in the analyses. If applicable, describe which groupings were chosen and why | 8-9 |
| Statistical methods | 12 | (*a*) Describe all statistical methods, including those used to control for confounding | 9 |
|  |  | (*b*) Describe any methods used to examine subgroups and interactions | 9 |
|  |  | (*c*) Explain how missing data were addressed | 7, 9 |
|  |  | (*d*) *Cohort study*—If applicable, explain how loss to follow-up was addressed  *Case-control study*—If applicable, explain how matching of cases and controls was addressed  *Cross-sectional study*—If applicable, describe analytical methods taking account of sampling strategy | 7-9 |
|  |  | (*e*) Describe any sensitivity analyses |  |
| Results | | Recommendation | Page  No |
| Participants | 13* | (a) Report numbers of individuals at each stage of study—eg numbers potentially eligible, examined for eligibility, confirmed eligible, included in the study, completing follow-up, and analysed | sF1 |
|  |  | (b) Give reasons for non-participation at each stage | sF1 |
|  |  | (c) Consider use of a flow diagram | sF1 |
| Descriptive data | 14* | (a) Give characteristics of study participants (eg demographic, clinical, social) and information on exposures and potential confounders | T1 |
|  |  | (b) Indicate number of participants with missing data for each variable of interest | T1 |
|  |  | (c) *Cohort study*—Summarise follow-up time (eg, average and total amount) | T2 |
| Outcome data | 15* | *Cohort study*—Report numbers of outcome events or summary measures over time | T2 |
|  |  | *Case-control study—*Report numbers in each exposure category, or summary measures of exposure |  |
|  |  | *Cross-sectional study—*Report numbers of outcome events or summary measures |  |
| Main results | 16 | (*a*) Give unadjusted estimates and, if applicable, confounder-adjusted estimates and their precision (eg, 95% confidence interval). Make clear which confounders were adjusted for and why they were included | T2 |
|  |  | (*b*) Report category boundaries when continuous variables were categorized | T2 |
|  |  | (*c*) If relevant, consider translating estimates of relative risk into absolute risk for a meaningful time period |  |
| Other analyses | 17 | Report other analyses done—eg analyses of subgroups and interactions, and sensitivity analyses | T2; sT2, sT3, sT6 |
| Discussion | |  | |
| Key results | 18 | Summarise key results with reference to study objectives | 12 |
| Limitations | 19 | Discuss limitations of the study, taking into account sources of potential bias or imprecision. Discuss both direction and magnitude of any potential bias | 15 |
| Interpretation | 20 | Give a cautious overall interpretation of results considering objectives, limitations, multiplicity of analyses, results from similar studies, and other relevant evidence | 13–15 |
| Generalisability | 21 | Discuss the generalisability (external validity) of the study results | 12-13 |
| Other information | |  | |
| Funding | 22 | Give the source of funding and the role of the funders for the present study and, if applicable, for the original study on which the present article is based | 16–17 |

s denotes supplementary; F, figure; T, table.

# **Supplementary Table 2: Incidence rates in proximal, mid or distal coronary arteries, unadjusted hazard ratios, and adjusted hazard ratios for plaque progression ≥50% luminal obstruction, or segment treated with PCI or CABG**

| **Segments (segment level)** |  | **N segments**  **at risk (%)** | **Follow-up (years)** | **KM Event rate** | **IR (95% CI)** | **HR (95% CI)**  **P** | **Adj HR (95% CI)**  **P** |
| --- | --- | --- | --- | --- | --- | --- | --- |
| Proximal RCA |  |  |  |  |  |  |  |
| NS |  | 95,501 (8.6%) | 598082 | 754 (2.6%) | 1.26 (1.17-1.35) | Reference;  *p=N/A* | Reference; *p=N/A* |
| FS |  | 63,330 (8.3%) | 376712 | 700 (4.1%) | 1.86 (1.73-2.00) | 1.49 (1.35-1.66);  *p<0.001* | 1.30 (1.17-1.44);  *p<0.001* |
| S |  | 31,307 (7.7%) | 206370 | 536 (5.1%) | 2.60 (2.39-2.83) | 2.00 (1.79-2.24);  *p<0.001* | 1.90 (1.69-2.14);  *p<0.001* |
| Middle RCA |  |  |  |  |  |  |  |
| NS |  | 95,473 (8.6%) | 597534 | 811 (3.0%) | 1.36 (1.27-1.45) | Reference;  *p=N/A* | Reference;  *p=N/A* |
| FS |  | 63,311 (8.3%) | 375890 | 838 (4.8%) | 2.23 (2.08-2.39) | 1.66 (1.51-1.83);  *p<0.001* | 1.42 (1.29-1.57);  *p<0.001* |
| S |  | 31,285 (7.7%) | 205594 | 688 (5.9%) | 3.25 (3.01-3.51) | 2.33 (2.11-2.59);  *p<0.001* | 1.99 (1.78-2.21);  *p<0.001* |
| Distal RCA |  |  |  |  |  |  |  |
| NS |  | 95,506 (8.6%) | 598904 | 488 (1.9%) | 0.81 (0.75–0.89) | Reference;  *p=N/A* | Reference;  *p=N/A* |
| FS |  | 63,341 (8.3%) | 377808 | 457 (2.8%) | 1.21 (1.10-1.33) | 1.51 (1.33-1.71);  *p<0.001* | 1.30 (1.14-1.48);  *p<0.001* |
| S |  | 31,330 (7.6%) | 207278 | 362 (3.6%) | 1.75 (1.58-1.94) | 2.07 (1.81-2.37);  *p<0.001* | 1.86 (1.61-2.15);  *p<0.001* |
| LMCA |  |  |  |  |  |  |  |
| NS |  | 103,365 (9.3%) | 651507 | 504 (1.6%) | 0.77 (0.71–0.84) | Reference;  *p=N/A* | Reference;  *p=N/A* |
| FS |  | 71,791 (9.4%) | 432603 | 455 (2.2%) | 1.05 (0.96-1.15) | 1.36 (1.20-1.55);  *p<0.001* | 1.18 (1.04-1.35);  *p=0.01* |
| S |  | 39,744 (9.8%) | 267123 | 224 (1.7%) | 0.84 (0.74–0.96) | 1.06 (0.90-1.24);  *p=0.49* | 1.03 (0.88-1.22)  *p=0.69* |
| Proximal LAD |  |  |  |  |  |  |  |
| NS |  | 79,746 (7.2%) | 494795 | 1249 (5.3%) | 2.52 (2.39-2.67) | Reference;  *p=N/A* | Reference; *p=N/A* |
| FS |  | 56,212 (7.4%) | 332055 | 1078 (7.0%) | 3.25 (3.06-3.45) | 1.30 (1.20-1.41);  *p<0.001* | 1.08 (0.99-1.18);  *p=0.06* |
| S |  | 30,495 (7.5%) | 200407 | 713 (7.3%) | 3.56 (3.31-3.83) | 1.37 (1.25-1.50);  *p<0.001* | 1.22 (1.11-1.35);  *p<0.001* |
| Middle LAD |  |  |  |  |  |  |  |
| NS |  | 79,712 (7.2%) | 493699 | 1438 (5.9%) | 2.91 (2.77-3.07) | Reference;  *p=N/A* | Reference;  *p=N/A* |
| FS |  | 56,207 (7.4%) | 331233 | 1209 (7.3%) | 3.65 (3.45-3.86) | 1.26 (1.17-1.36);  *p<0.001* | 1.06 (0.77-1.05);  *p=0.17* |
| S |  | 30,478 (7.5%) | 199667 | 812 (7.7%) | 4.07 (3.80-4.36) | 1.36 (1.25-1.48);  *p<0.001* | 1.24 (1.13-1.35);  *p<0.001* |
| Distal LAD |  |  |  |  |  |  |  |
| NS |  | 79,929 (7.2%) | 498904 | 392 (1.7%) | 0.76 (0.71–0.87) | Reference;  *p=N/A* | Reference;  *p=N/A* |
| FS |  | 56,370 (7.4%) | 335890 | 282 (1.9%) | 0.84 (0.75–0.94) | 1.08 (0.92-1.25);  *p=0.35* | 0.90 (0.77-1.05);  *p=0.18* |
| S |  | 30,577 (7.6%) | 202966 | 175 (1.9%) | 0.86 (0.74-1.00) | 1.06 (0.89-1.27);  *p=0.52* | 0.95 (0.79-1.15);  *p=0.60* |
| Proximal LCX |  |  |  |  |  |  |  |
| NS |  | 100,046 (9.0%) | 627397 | 965 (3.3%) | 1.54 (1.44-1.64) | Reference;  *p=N/A* | Reference;  *p=N/A* |
| FS |  | 68,531 (9.0%) | 409863 | 811 (4.3%) | 1.98 (1.85-2.12) | 1.30 (1.18-1.43);  *p<0.001* | 1.10 (1.00-1.21);  *p=0.04* |
| S |  | 37,112 (9.2%) | 247385 | 574 (4.4%) | 2.32 (2.14-2.52) | 1.47 (1.32-1.63);  *p<0.001* | 1.36 (1.22-1.52);  *p<0.001* |
| Distal LCX |  |  |  |  |  |  |  |
| NS |  | 100,099 (9.0%) | 629589 | 506 (1.8%) | 0.80 (0.74–0.88) | Reference;  *p=N/A* | Reference;  *p=N/A* |
| FS |  | 68,558 (9.0%) | 411233 | 486 (2.7%) | 1.18 (1.08-1.29) | 1.49 (1.31-1.68);  *p<0.001* | 1.25 (1.10-1.42);  *p=0.04* |
| S |  | 37,135 (9.2%) | 248291 | 384 (3.2%) | 1.55 (1.40-1.71) | 1.86 (1.63-2.12);  *p<0.001* | 1.65 (1.43-1.89);  *p<0.001* |
| First diagonal |  |  |  |  |  |  |  |
| NS |  | 77,825 (7.0%) | 483288 | 672 (2.6%) | 1.39 (1.29-1.50) | Reference;  *p=N/A* | Reference;  *p=N/A* |
| FS |  | 54,790 (7.2%) | 323836 | 558 (3.3%) | 1.72 (1.58-1.87) | 1.25 (1.12-1.40);  *P=<0.001* | 1.04 (0.92-1.16);  *p=0.55* |
| S |  | 29,712 (7.4%) | 195820 | 389 (4.0%) | 1.99 (1.80-2.19) | 1.39 (1.23-1.58);  *p<0.001* | 1.22 (1.07-1.40);  *p=0.003* |
| First Obtuse Marginal | | |  |  |  |  |  |
| NS |  | 98,173 (8.9%) | 615068 | 824 (2.6%) | 1.34 (1.25-1.43) | Reference;  *p=N/A* | Reference;  *p=N/A* |
| FS |  | 66,947 (8.8%) | 400000 | 694 (3.9%) | 1.74 (1.61–1.87) | 1.31 (1.18-1.45);  *p<0.001* | 1.11 (1.00-1.23);  *p<0.05* |
| S |  | 35,925 (8.9%) | 239169 | 452 (4.0%) | 1.89 (1.72-2.07) | 1.37 (1.22-1.54);  *p<0.001* | 1.22 (1.08-1.38);  *p=0.001* |
| PDA/LPD |  |  |  |  |  |  |  |
| NS |  | 102,387 (9.2%) | 644384 | 629 (2.1%) | 0.98 (0.90-1.06) | Reference;  *p=N/A* | Reference;  *p=N/A* |
| FS |  | 71,047 (9.3%) | 427671 | 455 (2.4%) | 1.06 (0.97-1.17) | 1.10 (0.97-1.24);  *p=0.14* | 0.92 (0.81-1.04);  *p=0.17* |
| S |  | 39,221 (9.7%) | 263133 | 346 (2.4%) | 1.31 (1.18-1.46) | 1.31 (1.15-1.50);  *p<0.001* | 1.19 (1.04-1.37);  *p=0.01* |

Table depicting incidence rates per 1000-segment years, unadjusted hazard ratio, and adjusted hazard ratios, with 95% confidence intervals for progressive lesions ≥50% luminal obstruction at segment level. CABG indicates coronary artery bypass grafting; IR, incidence rate; HR, hazard ratio; Adj, adjusted; P, p-value; CI, confidence interval; NS, non-smokers; FS, former smokers; S, current smokers; RCA, right coronary artery; LAD, left anterior descending artery; LCX, left circumflex artery; PDA, posterior descending artery; LPD, left posterior descending artery.

# **Supplementary Table 3: Primary outcomes stratified by sex, indication, and risk factors: Incidence rates for new coronary artery lesions defined as ≥50% luminal stenosis, or lesion treated with CABG or PCI**

|  |  | **Follow-up (Person-year)** | **KM Event rate** | **IR (95% CI)** | **HR (95% CI);**  **P** | **Adj HR (95% CI);**  **P** |
| --- | --- | --- | --- | --- | --- | --- |
| **Sex** |  |  |  |  |  |  |
| Men |  |  |  |  |  |  |
| NS |  | 339178 | 3280 (16.4%) | 9.7 (9.3–10.0) | Reference;  *p=N/A* | Reference;  *p=N/A* |
| FS |  | 272654 | 3333 (19.8%) | 12.2 (11.8-12.6) | 1.27 (1.21-1.33);  *p<0.001* | 1.18 (1.12-1.24);  *p<0.001* |
| S |  | 157581 | 2097 (20.9%) | 13.3 (12.7-13.9) | 1.36 (1.29-1.44);  *p<0.001* | 1.30 (1.23-1.38);  *p<0.001* |
| Women |  |  |  |  |  |  |
| NS |  | 293973 | 1591 (8.8%) | 5.4 (5.2–5.7) | Reference;  *p=N/A* | Reference;  *p=N/A* |
| FS |  | 144020 | 914 (11.0%) | 6.3 (5.9–6.8) | 1.18 (1.08-1.28);  *p<0.001* | 1.15 (1.05-1.25);  *p<0.001* |
| S |  | 97378 | 780 (12.7%) | 8.0 (7.5–8.6) | 1.47 (1.35-1.60);  *p<0.001* | 1.50 (1.36-1.64);  *p<0.001* |
| Women-to-men^a^ |  |  |  |  | 1.08 (0.97-1.19);  *p=0.16* | 1.08 (0.97-1.20);  *p=0.16* |
| **Indication**  **at follow-up** |  |  |  |  |  |  |
| CCS |  |  |  |  |  |  |
| NS |  | 633151 | 1333 (3.6%) | 2.1 (2.0–2.2) | Reference;  *p=N/A* | Reference;  *p=N/A* |
| FS |  | 416712 | 1245 (5.1%) | 3.0 (2.8–3.2) | 1.42 (1.31-1.53);  *p<0.001* | 1.24 (1.14-1.34);  *p<0.001* |
| S |  | 254959 | 637 (4.1%) | 2.5 (2.3–2.7) | 1.18 (1.07-1.30);  *p=0.001* | 1.05 (0.95-1.16);  *p=0.35* |
| ACS |  |  |  |  |  |  |
| NS |  | 633151 | 2890 (8.1%) | 4.6 (4.4–4.7) | Reference;  *p=N/A* | Reference;  *p=N/A* |
| FS |  | 416712 | 2438 (10.5%) | 5.9 (5.6–6.1) | 1.29 (1.22-1.36);  *p<0.001* | 1.12 (1.06-1.19);  *P<0.001* |
| S |  | 254959 | 1954 (12.4%) | 7.7 (7.3–8.0) | 1.66 (1.57-1.76);  *p<0.001* | 1.52 (1.44-1.62);  *P<0.001* |
| Other |  |  |  |  |  |  |
| NS |  | 633151 | 648 (1.8%) | 1.0 (0.9–1.1) | Reference;  *p=N/A* | Reference;  *p=N/A* |
| FS |  | 416712 | 564 (2.3%) | 1.4 (1.2–1.5) | 1.33 (1.19-1.49);  *p<0.001* | 1.17 (1.04-1.32);  *p*=0.007 |
| S |  | 254959 | 286 (2.2%) | 1.1 (0.9–1.3) | 1.08 (0.94-1.25); *p=0.26* | 1.13 (0.97-1.31);  *p=0.11* |
| **Risk factors** |  |  |  |  |  |  |
| Hypertension |  |  |  |  |  |  |
| NS |  | 273598 | 2515 (15.1%) | 9.2 (8.8–9.6) | Reference;  p=N/A | Reference;  p=N/A |
| FS |  | 20092 | 2300 (18.9%) | 11.4 (11.0-11.9) | 1.25 (1.18-1.32);  p<0.001 | 1.15 (1.09-1.22);  p=<0.001 |
| S |  | 85521 | 1060 (19.4%) | 12.4 (11.7-13.2) | 1.34 (1.25-1.44);  p<0.001 | 1.27 (1.18-1.37);  p<0.001 |
| Hyperlipidemia |  |  |  |  |  |  |
| NS |  | 367671 | 3579 (15.8%) | 9.7 (9.2–10.1) | Reference;  p=N/A | Reference;  p=N/A |
| FS |  | 274521 | 3258 (19.2%) | 11.9 (11.5-12.3) | 1.22 (1.17-1.28);  p<0.001 | 1.14 (1.09-1.20);  p<0.001 |
| S |  | 183267 | 2319 (19.8%) | 12.7 (12.1-13.2) | 1.29 (1.23-1.36);  p<0.001 | 1.20 (1.14-1.27);  p<0.001 |
| Diabetes |  |  |  |  |  |  |
| NS |  | 69473 | 945 (24.0%) | 13.6 (12.8-14.5) | Reference;  p=N/A | Reference;  p=N/A |
| FS |  | 55279 | 911 (26.9%) | 16.5 (15.4-17.6) | 1.22 (1.12-1.34);  p<0.001 | 1.14 (1.04-1.25);  p=0.005 |
| S |  | 24859 | 443 (27.4%) | 18.7 (16.2-19.6) | 1.30 (1.16-1.46);  p<0.001 | 1.21 (1.08-1.36);  p=0.001 |
| Prior CAD |  |  |  |  |  |  |
| NS |  | 273973 | 3572 (19.5%) | 13.0 (12.6-13.5) | Reference;  p=N/A | Reference;  p=N/A |
| FS |  | 206114 | 3185 (22.7%) | 15.5 (14.9-16.0) | 1.19 (1.13-1.24);  p<0.001 | 1.15 (1.09-1.20);  p=<0.001 |
| S |  | 162632 | 2302 (21.3%) | 14.2 (13.6-14.7) | 1.08 (1.03-1.14);  p=0.003 | 0.99 (0.94-1.05);  p=0.90 |

Values are expressed as number. Table depicting incidence rates per 1000-patient years, unadjusted hazard ratio, and adjusted hazard ratios, with 95% confidence intervals for progressive lesions ≥50% luminal obstruction at patient level. Indication is based on indication at angiogram detecting the progressive lesion. CABG indicates coronary artery bypass grafting; PCI, percutaneous coronary intervention; IR, incidence rate; HR, hazard ratio; Adj, adjusted; P, p-value; CI, confidence interval; CAD, coronary artery disease; NS, non-smokers; FS, former smokers; S, current smokers.

^a^=Unadjusted and adjusted women-to-men hazard ratios.

# **Supplementary Table 4: Mean number of coronary angiographies by smoking status**

| **Mean number of coronary angiographies* by smoking status** | |
| --- | --- |
| **Non-smokers** | 1.24 (0.65) |
| **Former smokers** | 1.28 (0.72) |
| **Current smokers** | 1.32 (0.80) |
| **Total** | 1.27 (0.70) |
| *Includes index coronary angiography | |

#

# **Supplementary Table 5: Patients divided by numbers of follow-up angiographies performed and smoking status**

| **n angio** | | **Non-smokers** | **Former smokers** | **Current smokers** | **TOTAL** |
| --- | --- | --- | --- | --- | --- |
| 0 | 86,286 (86.2%) | | 60,582 (84.2%) | 32,825 (82.4%) | 182,693 (84.8%) |
| 1 | 10,517 (10.2%) | | 8,180 (11.4%) | 4,844 (12.2%) | 23,541 (10.9%) |
| 2 | 2,480 (2.4%) | | 2,069 (2.9%) | 1,304 (3.3%) | 5,853 (2.7%) |
| 3 | 798 (0.8%) | | 661 (0.9%) | 497 (1.3%) | 1,956 (0.9%) |
| ≥4 | 503 (0.5%) | | 472 (0.7%) | 346 (0.9%) | 1,321 (0.6%) |
| TOTAL | 103,584 (100%) | | 71,964 (100%) | 39,816 (100%) | 215,364 (100%) |

#

# **Supplementary Table 6: Smoking status at life-time last follow-up angiography divided by smoking status at baseline angiography**

|  |  | **Last follow-up smoking status** | | |  |
| --- | --- | --- | --- | --- | --- |
|  |  | NS | FS | S | Total |
| **Baseline smoking status** | |  |  |  |  |
| NS |  | 10,721 (80.0%) | 2,667 (19.6%) | 190 (1.4%) | 13,578 (100%) |
| FS |  | 2,103 (19.2%) | 8,350 (76.3%) | 494 (4.5%) | 10,947 (100%) |
| S |  | 497 (7.3%) | 3,001 (44.3%) | 3,280 (48.4%) | 6,778 (100%) |
| Total |  | 13,321 (42.6%) | 14,018 (44.8%) | 3,964 (12.7%) | 31,303 (100%) |

The table showcase smoking status at life-time last angiography for all patients undergoing one or more follow-up angiographies divided by smoking status at index angiography. This demonstrates how smoking status generally is changed through-out the follow-up period and secondary prevention. NS indicates non-smokers, FS; former smokers, and S; current smokers.

#

# **Supplementary Table 7: Median time to angiography detecting progressive lesion on patient level**

| **Non-smokers** | 4.5 (1.8, 7.7) |
| --- | --- |
| **Former smokers** | 4.1 (1.6, 7.4) |
| **Current smokers** | 4.9 (2.0, 8.2) |
| **Total** | 4.5 (1.8, 7.2) |

Numbers indicate years (lower, upper quartile).

# **Supplementary Table 8: Adjusted relative risk of progression in major coronary arteries compared with the right coronary artery**

| **Former smokers** |  | **HR (95% CI)** | **P** |
| --- | --- | --- | --- |
|  | LAD | 0.82 (0.74–0.90) | <0.001 |
|  | LCX | 0.90 (0.80–0.99) | 0.05 |
|  | LM | 0.91 (0.78–1.05) | 0.2 |
|  | Branches | 0.83 (0.75–0.92) | <0.001 |
| **Current smokers** |  |  |  |
|  | LAD | 0.63 (0.57–0.70) | <0.001 |
|  | LCX | 0.77 (0.69–0.87) | <0.001 |
|  | LM | 0.51 (0.43–0.61) | <0.001 |
|  | Branches | 0.66 (0.59–0.74) | <0.001 |

Interaction term analysis comparing the adjusted hazard ratio of former and current smokers to non-smokers of the right coronary artery (reference artery) to the adjusted hazard ratio of former and current smokers of the other major arteries.

# **Supplementary Figure 1: Inclusion and exclusion flow chart**


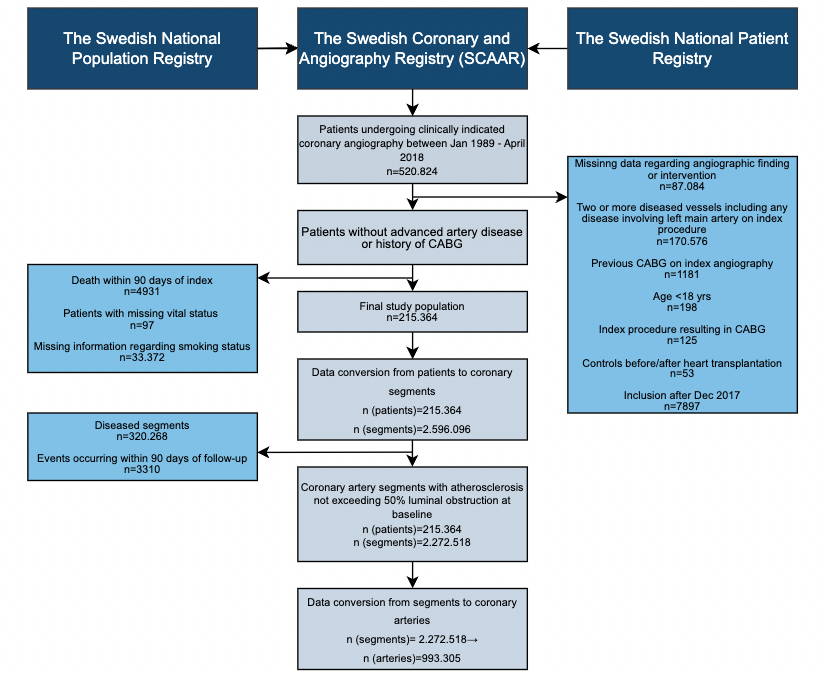


# **Supplementary Figure 2: Total distribution of progression lesions for each smoking-cohort**

**
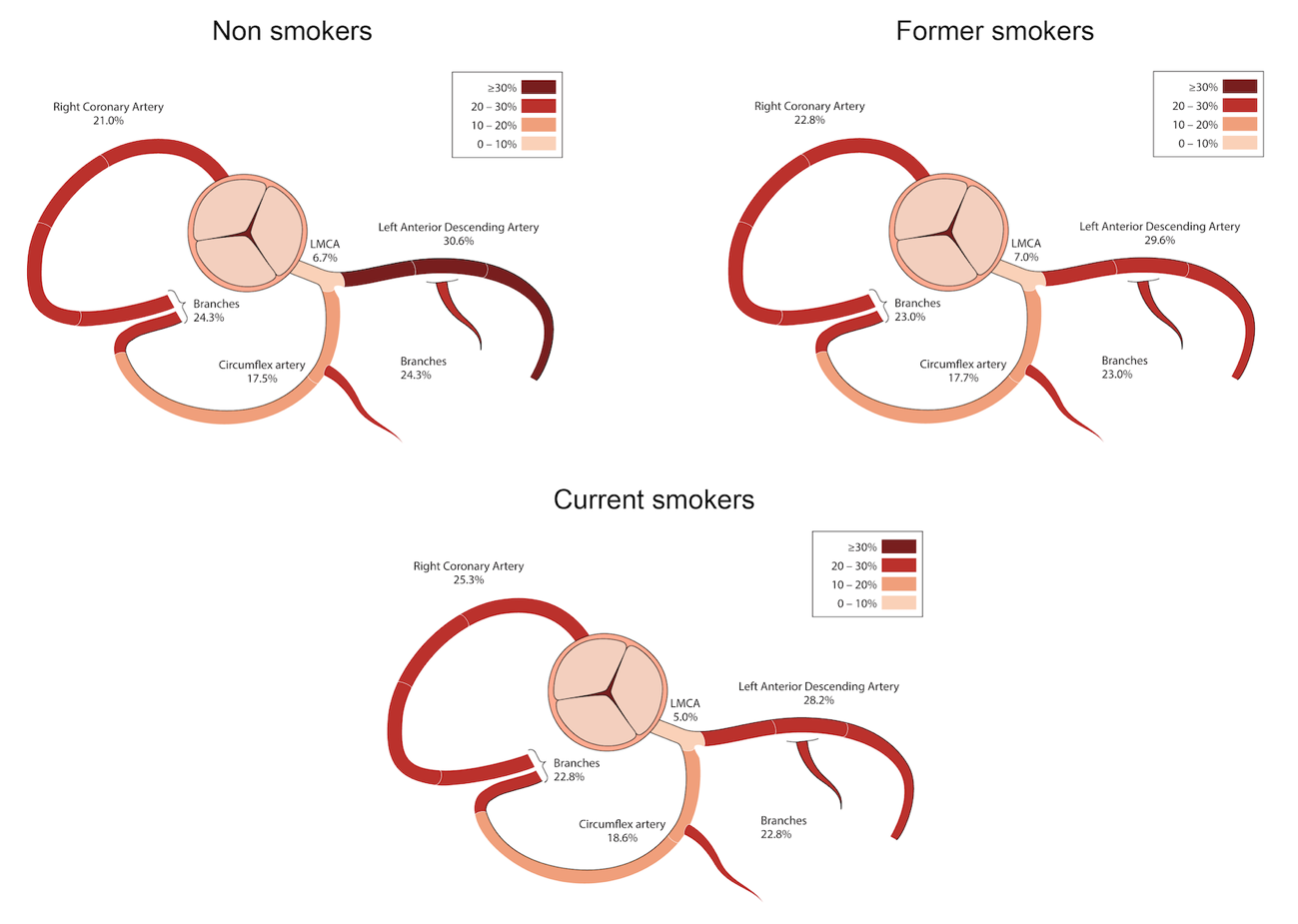
**

Supplementary Figure 1 depicts the distribution of the progressive lesions at the artery level for (A) non-smokers, (B) former smokers, and (C) current smokers. % for each major vessel headline sums to 100% within each diagram. LAD indicates left anterior descending artery; LMCA, left main coronary artery; LCX, left circumflex artery; RCA, right coronary artery; Branches include first diagonal artery, first obtuse marginal artery, and posterior descending/left posterior descending artery.
